# Reticulospinal modulation of muscle activation and electromechanical coupling: evidence from the StartReact paradigm

**DOI:** 10.3389/fnhum.2025.1610211

**Published:** 2025-05-21

**Authors:** Antonia Maria Eilfort, Linard Filli

**Affiliations:** ^1^Spinal Cord Injury Center, Balgrist University Hospital, Zurich, Switzerland; ^2^Neuroscience Center Zurich, University of Zurich, Zurich, Switzerland; ^3^Department of Health Science and Technology, ETH Zurich, Zurich, Switzerland; ^4^Swiss Center for Movement Analysis (SCMA), Balgrist Campus AG, Zurich, Switzerland

**Keywords:** electromechanical delay (EMD), descending motor pathways, corticospinal system, neuromotor control, motor systems, subcortical motor control

## Abstract

**Introduction:**

Movement initiation relies on descending motor drive conveying motor commands from the brain to spinal motor circuits, leading to the activation of specific muscles to produce the intended movement. While the role of descending motor drive on the onset of muscle activation has been extensively examined, its impact on motor unit recruitment, muscle fiber activation, and the electromechanical delay (EMD) remain poorly understood. This study aimed to elucidate the role of the reticulospinal (RS) system in shaping muscle activation patterns, movement initiation, and the EMD by employing the StartReact paradigm.

**Methods:**

The StartReact paradigm was implemented in 29 healthy participants performing 14 single-joint motor tasks including both upper and lower extremities. Muscle activity was recorded using surface electromyography (EMG), while movement patterns were acquired via motion capture technology. Muscle activation and movement patterns were analyzed in both temporal and amplitude domains to characterize differences between movements cued by either loud (LAS: 120 dB) or moderate acoustic stimuli (MAS: 82 dB). EMD was defined as the time interval between EMG onset and movement initiation.

**Results:**

Our results revealed faster and more pronounced muscle activation and movement performance in response to LAS compared to MAS. Notably, EMD was significantly reduced in LAS trials, suggesting that enhanced RS drive facilitates more rapid electromechanical coupling.

**Discussion:**

These findings suggest that RS drive not only shortens muscular reaction times – characteristic of the StartReact effect - but also modulates muscle activation and movement dynamics in a way that accelerates the transition from muscle activation to movement. The observed reduction in EMD likely reflects changes in motor unit recruitment and muscle fiber activation, highlighting an additional mechanism through which the RS system enables rapid, explosive motor responses. This study provides novel insights into how descending motor drive modulates muscle activation and movement execution, and emphasizes the relevance of the RS system in supporting rapid, high-force movements essential for protective reflexes and athletic performances.

## 1 Introduction

The electromechanical delay (EMD) refers to the time interval between the onset of electrical activity in a muscle and the generation of measurable motion or force (Norman and Komi, 1979; Cano et al., 2024; Cavanagh and Komi, 1979; Cè et al., 2013). EMD physiology can be subdivided into two major components characterized by electrochemical and mechanical processes. The electrochemical component of the EMD encompasses the processes from the arrival of electrical activity at the muscle to the initiation of motion. This includes synaptic transmission of the arriving action potential and its conduction along muscle fibers and T-tubules (Cè et al., 2013). This process triggers calcium release from the sarcoplasmic reticulum, which binds to troponin, triggering cross-bridge cycling and the initial power strokes of muscular contraction (Cè et al., 2013). Once muscle contraction is initiated, the mechanical component of the EMD is responsible for the development of tension within the muscle fibers. This tension must overcome the compliance of connective tissues, such as tendons, before force is transmitted to the skeletal system, resulting in measurable motion.

The EMD is influenced by numerous factors such as action potential propagation, excitation-contraction coupling, and muscle force transmission along the series elastic component including the types of muscle fibers recruited (Nordez et al., 1970; Hill, 1949). Different factors, such as fatigue (Cè et al., 2013; Castellote et al., 2017), temperature (Cè et al., 2013), mechanical stress (Toninelli et al., 2024; Esposito et al., 2011), contraction intensity (Cè et al., 2013; Zhou et al., 1995), as well as exercise (Grosset et al., 2009), particularly exercise-induced muscle damage (Howatson, 2010), have been reported to modulate EMD. However, the role of descending motor drive on EMD remains largely unexplored. Descending motor pathways convey information from the brain to spinal motoneurons (either directly or via spinal interneurons), governing different types of movements. Exploring how variations in descending motor system activity affect EMD will provide valuable insights into the neuromuscular mechanisms underlying movement control.

The corticospinal (CS) and reticulospinal (RS) systems are the two primary descending motor systems conveying motor commands to the spinal cord (Lemon, 2008). The CS system is a phylogenetically new motor system crucial for voluntary and precise motor control, particularly in executing dexterous movements (Lemon, 2008; Baker et al., 2015). In contrast, the RS system is an evolutionary conserved motor system that controls fundamental motor functions, including locomotion, posture, and force control (Baker et al., 2015; Lawrence and Kuypers, 1968; Glover and Baker, 2020). Additionally, the RS system plays a key role in mediating rapid, reflexive motor responses, such as postural adjustments and express visuomotor responses (Deliagina et al., 2014; Glover and Baker, 2019). Given their distinct roles, the CS and RS systems may mediate movements differently with regard to motor unit activation and muscle fiber recruitment, potentially leading to varied effects on muscle activation and EMD.

In this study, we assessed the influence of RS motor drive on EMD characteristics. The StartReact paradigm was employed to 29 healthy volunteers executing 14 tasks involving both the upper and lower extremities. The StartReact effect is characterized by an accelerated muscle activation (i.e., premotor reaction time) when movement initiation is paired with a loud acoustic stimulus (LAS) (Valls-Solé et al., 1995; Valls-Solé et al., 2008). Robust evidence suggests that this accelerated muscle activation reflects enhanced RS drive and that the StartReact is a valid biomarker of RS contributions to movements in humans (Tapia et al., 2022; Nonnekes et al., 2014). Whereas the impact of RS drive on electrical muscle activation has been well documented, its potential influence on the EMD remains unclear. By assessing both muscular and kinematic responses to the StartReact paradigm, we aim to elucidate the role of the RS system in movement control, from initial muscle activation through to the initiation of movement, including the EMD. A deeper understanding of how the RS system shapes both muscle activation and movement initiation is essential to advancing our knowledge of RS contributions to movement control.

## 2 Methods

### 2.1 Paradigm

Twenty-nine healthy participants (26.17 ± 3.49 years, 19 females) were included in the study. Written informed consent was obtained from all participants. The study was approved by the Ethics Committee of Canton Zurich (Study ID: 2021-00973) and was conducted according to the declaration of Helsinki.

The study consisted of two visits for each participant, 14 days apart. One visit was dedicated to StartReact in the upper extremities and one to StartReact in the lower extremities. Findings on the StartReact paradigm and detailed methodological descriptions of the experimental procedures have been reported previously (Eilfort et al., 2025). First, participants were familiarized with LAS (120 dB, 50 ms, 1,000 Hz), each LAS being preceded by a warning stimulus (WS; 92 dB, 50 ms, 500 Hz). After familiarization, participants underwent seven blocks of StartReact per visit. Each block consisted of 30 imperative stimuli (20 moderate acoustic stimuli (MAS; 82 dB, 50 ms, 1,000 Hz) and 10 LAS), in a randomized order. The imperative stimuli were preceded by WS, with the interstimulus intervals (1.5–3 s) and the inter-trial intervals (6–10 s) being pseudorandomized to minimize stimulus anticipation. Participants were instructed to perform a specific movement as fast as possible after the imperative stimulus.

Each participant performed one block each for seven upper extremity tasks (shoulder extension and flexion, elbow extension and flexion, wrist extension and flexion, and finger abduction) and seven lower extremity tasks (hip extension and flexion, knee extension and flexion, ankle plantar flexion and dorsal flexion, and toe extension).

### 2.2 Data collection

Muscle activity was recorded by surface electromyography (sEMG) from the relevant muscles for each task (deltoideus pars spinalis, deltoideus pars clavicularis, triceps brachii, biceps brachii, extensor digitorum, flexor carpi radialis, first dorsal interosseus, gluteus maximus, quadriceps rectus femoris, quadriceps vastus medialis, semitendinosus, gastrocnemius, tibialis anterior, extensor hallucis brevis). sEMG was recorded with bipolar Ag-AgCl surface EMG electrodes (H124SG, Kendall) and sampled at 2000 Hz by a wireless EMG system (Myon Aktos, Cometa Systems, Bareggio, Italy).

In addition, reflective motion capture markers were placed on anatomical landmarks, seven for upper extremity tasks and eight for lower extremity tasks. Motion capture was sampled by a 27-camera optical motion capturing system (Vicon UK) at 200 Hz. EMG, acoustic stimuli, and motion capture were time synchronized (Vicon Nexus).

### 2.3 Data analysis

Data preparation was performed in Matlab (Matlab R2024a, Mathworks Inc. Natick, United States) and plots using the *ggplot2* package (v 3.5.1) of R (version 4.4.0) and Rstudio (2024.04.1).

EMG data were bandpass filtered (10–500 Hz) and rectified. The onset for target muscles were determined as the EMG activity that exceeded the baseline mean by two standard deviations, the baseline was calculated as the mean EMG activity within a window of 100 ms before the imperative stimulus (LAS or MAS). Mean EMG traces over all participants for each target muscle were plotted over 50 ms before and 200 ms after muscle onset. The root mean square (RMS), peak amplitude, and time of peak amplitude were calculated for mean EMG data per participant and task over 50 ms before and 200 ms after muscle onset. Median values and interquartile ranges (IQR) for all three measures (RMS, peak amplitude, time of peak) were calculated for each task and overall tasks.

Movement onsets were determined as the first point with *>* 0.2° angular displacement from the starting position at stimulus presentation (Valls-Solé et al., 1995). Mean kinematic traces over all participants for each target muscle were plotted over 0 ms to 500 ms after movement onset. The RMS, peak amplitude, and time of peak amplitude were calculated for mean kinematics data per participant and task over 0 ms to 500 ms after movement onset. Median values and IQR for all three measures (RMS, peak amplitude, time of peak) were calculated for each task and overall tasks.

EMG and kinematic reaction times were calculated as the time from stimulus presentation to muscle or movement onset. The difference between movement reaction times and muscle reaction times was calculated and referred to as the EMD. The difference between EMD after LAS and MAS were calculated.

### 2.4 Statistics

Statistical analysis was conducted with R (version 4.4.0) and Rstudio (2024.04.1) using the lmer function of the lme4 package for fitting linear mixed-effects models. Post hoc pairwise comparisons of estimated marginal means (least-squares means) were conducted using the emmeans package (v 1.10.2) and Bonferroni corrected. The significance level was set at = 0.05 for all tests.

For both EMG and kinematic data, we assessed the effect of the imperative stimulus (LAS vs. MAS) on RMS, peak amplitude, and time of peak amplitude, as well as the EMD by fitting linear mixed effect models with stimulus type as the fixed effect and task as the random effect. An analysis of variance (ANOVA) of the models was performed. We additionally analyzed the effect of task and type and their interaction by fitting models with stimulus type and task as the fixed effects and subject as a random effect. An ANOVA was performed on the fitted models. Post hoc tests were performed to assess the effect of the imperative stimulus (LAS vs. MAS) on each task for all parameters. Additionally, the effect of tasks on the difference between EMD of MAS and LAS trials was also assessed with a linear mixed effect model with the task as a fixed effect and the subject as a random effect.

## 3 Results

### 3.1 EMG activity patterns differ between ballistic movements cued by LAS vs. MAS

**Figure 1:**
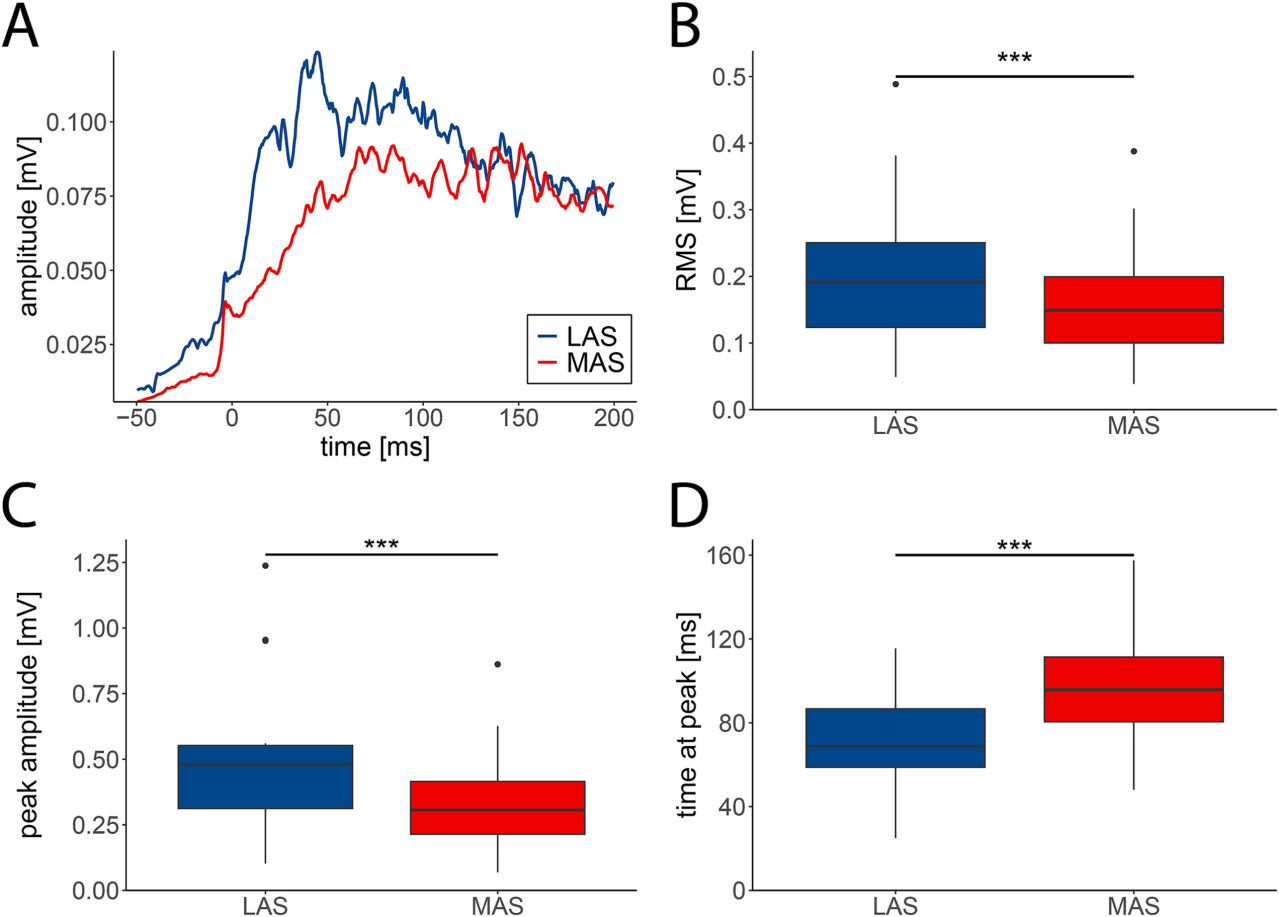
EMG activity patterns in response to loud (LAS; blue) and moderate acoustic stimuli (MAS; red). **(A)** Grand average EMG trace (*n* = 29 participants) of the hip extensor muscle from −50 to 200 ms relative to muscle onset. **(B)** The averaged root mean square (RMS) of EMG signals was significantly enhanced in LAS compared to MAS trials. **(C)** EMG amplitudes were significantly greater in LAS vs. MAS trials. **(D)** Latencies of peak EMG amplitudes were shorter in LAS compared to MAS trials. **B–D** depict medians +/− interquartile ranges of all tasks (*n* = 14) from −50 to 200 ms relative to muscle onset (***p < 0.001).

Muscle activity during movement initiation typically revealed stronger EMG responses in LAS vs. MAS trials. This was evident on the single muscle level (Supplementary Figure 1A), such as for the hip extensor muscle (Figure 1A). The averaged RMS across all 14 muscles from −50 to 200 ms relative to EMG onsets revealed EMG signals that were significantly enhanced in LAS (median = 0.18 mV, IQR = 0.20 mV) compared to MAS trials (median = 0.14 mV, IQR = 0.18 mV), with repeated-measures ANOVA revealing a significant effect of stimulus type (LAS vs. MAS) on RMS (F(1,13) = 16.76, *p <* 0.001; Figure 1B). Similarly, the averaged peak amplitude across all muscles was higher in response to LAS (median = 0.47 mV, IQR = 0.50 mV) than MAS (median = 0.31 mV, IQR = 0.38 mV; F(1,13) = 36.82, *p <* 0.001; Figure 1C). Additionally, EMG peak amplitudes emerged earlier after EMG onset upon LAS (median = 69 ms, IQR = 73.38 ms) vs. MAS (median = 91.5 ms, IQR = 76.38 ms; F(1,13) = 21.5, *p <* 0.001; Figure 1D). Therefore, LAS triggered movements with faster and stronger muscle activation compared to movements cued by MAS.

Post-hoc analysis demonstrated that LAS-related increases in RMS were restricted to upper extremity muscles including shoulder, elbow, and finger muscles, but were absent for lower extremity muscles (Supplementary Figure 1B). Enhanced EMG peak amplitudes in response to LAS vs. MAS were observed for the majority of upper and lower extremity muscles, except for hip extensors and flexors, and knee extensors (Supplementary Figure 1C). Accelerated muscle activation in response to LAS vs. MAS was significant for the shoulder flexor, finger abductor, hip flexor and extensor, knee extensor, and ankle plantar flexor (Supplementary Figure 1D). Data on EMG reaction times and the StartReact effect are reported in a previous publication (Eilfort et al., 2025).

### 3.2 Differential movement dynamics in response to LAS vs. MAS

**Figure 2:**
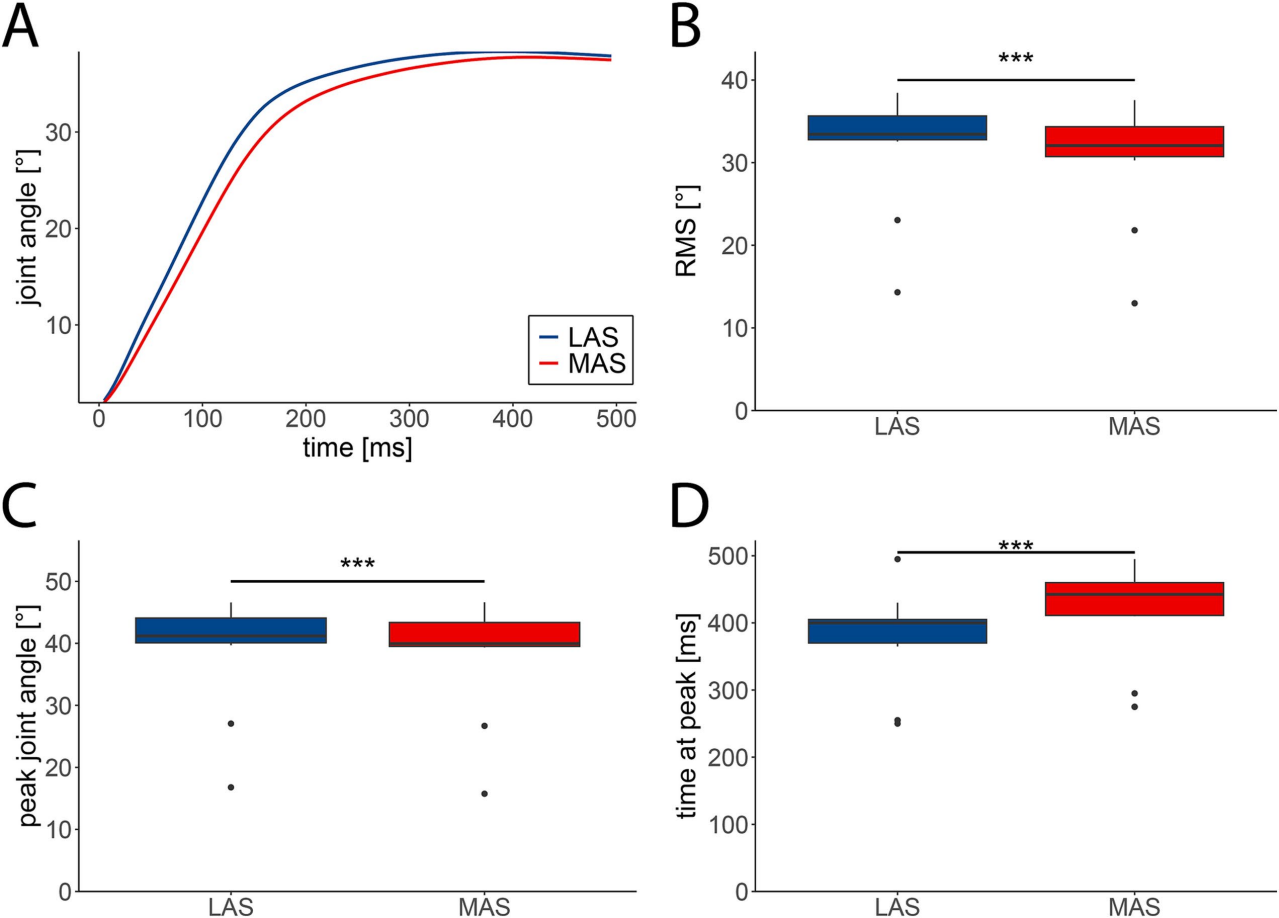
Kinematic characteristics of movements cued by loud (LAS; blue) and moderate acoustic stimuli (MAS; red). **(A)** Grand average angular displacement (*n* = 29 participants) of the hip during extension from 0 to 500 ms relative to movement onset. **(B)** The averaged root mean square (RMS) of angular displacements was significantly enhanced in LAS compared to MAS trials. **(C)** The averaged peak amplitude of angular displacements was significantly greater in LAS vs. MAS trials. **(D)** Latencies of peak angles were shorter in LAS compared to MAS trials. **B–D** depict medians +/− interquartile ranges of all tasks (*n* = 14) from 0 to 500 ms relative to movement onset (***p < 0.001).

Movement dynamics differed between LAS and MAS trials, with LAS trials leading to increased movement acceleration and amplitude. This pattern was evident for hip extension (Figure 2A) and most other assessed tasks (Supplementary Figure 2A). The averaged RMS of joint angles from 0 to 500 ms relative to movement onset across all 14 tasks were marginally higher in LAS (median = 33.75°, IQR = 8.15°) compared to MAS trials (median = 32.27°, IQR = 7.69°; F(1,13) = 103.39, *p <* 0.001; Figure 2B). Likewise, peak movement amplitudes were slightly greater in response to LAS (median = 40.99°, IQR = 10.03°) compared to MAS (median = 40.24^°^, IQR = 10.03°; F(1,13) = 49.9, *p <* 0.001; Figure 2C). Additionally, movement peak amplitudes occurred earlier in LAS (median = 390 ms, IQR = 130 ms) than MAS trials (median = 425 ms, IQR = 145 ms; F(1,13) = 68, *p <* 0.001; Figure 2D). Summarized, movement amplitudes were marginally higher and occurred earlier in LAS compared to MAS trials.

Post-hoc analysis revealed enhanced ankle joint RMS for the shoulder extensor and flexor, wrist extensor and flexor, knee extensor and flexor, and toe extensor (Supplementary Figure 2B). In contrast, task-specific differences between LAS vs. MAS in peak joint angles (Supplementary Figure 2C) and time at peak (Supplementary Figure 2D) were absent.

### 3.3 Altered electromechanical coupling in movements cued by LAS vs. MAS

**Figure 3:**
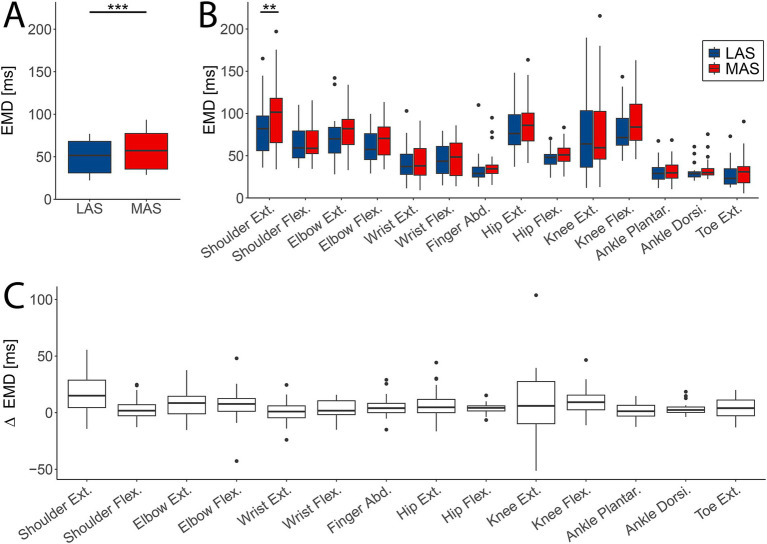
Difference in electromechanical delay (EMD) between loud (LAS; blue) and moderate acoustic stimuli (MAS; red). **(A)** The averaged EMD over all tasks (*n* = 14) was significantly shortened in LAS compared to MAS trials. **(B)** EMD values for every task and MAS and LAS trials for all participants (*n* = 29). Shoulder extension showed a significant difference in EMD between LAS and MAS trials. **(C)** Difference in EMD between LAS and MAS trials across subjects (*n* = 29) and tasks (**p < 0.01; ***p < 0.001).

The average EMD across all tasks was significantly reduced in response to LAS (median = 51.5 ms, IQR = 37.1 ms) compared to MAS (median = 57.3 ms, IQR = 41.9 ms; F(1,13) = 28.602, *p <* 0.001; Figure 3A). This effect remained significant when adding tasks as fixed effect (F(1,752.05) = 13.13, *p <* 0.001; Figure 3B). Additionally, EMD varied significantly between tasks (F(13,752.22) = 48.61, *p <* 0.001; Figure 3B), while the effect of stimulus type (LAS vs. MAS) was not task-dependent, as indicated by a non-significant interaction effect (F(31,752.05) = 0.49, *p* = 0.93; Figure 3B).

Post-hoc analysis revealed a significantly reduced EMD in the shoulder extension upon LAS vs. MAS (LAS: median = 82 ms, IQR = 41 ms vs. MAS: median = 101 ms, IQR = 52 ms; estimate = −18.20, SE = 6.38, z = −2.852, *p* = 0.0045; Figure 3B), whereas the other tasks did not reveal significant differences in EMD.

Additionally, delta EMD (difference in EMD between MAS and LAS trials) significantly differed between tasks (F(13,360.44) = 3.286, *p* < 0.001; Figure 3C), supporting the evidence reported above (Figure 3B). Overall, EMD was shorter in LAS compared to MAS trials, with variations observed across tasks. However, no significant interaction between stimulus type and task was found.

## 4 Discussion

Movement initiation is influenced by two key physiological processes: the electrical muscle activation and the initiation of movement generated by muscle contraction. While the impact of the RS system on premotor reaction time (i.e., electrical muscle activation) has been extensively investigated using the StartReact paradigm (Valls-Solé et al., 1995; Carlsen and Maslovat, 2019), its influence on electromechanical coupling remains poorly understood. Here, we examined the effects of descending motor drive on muscle activation patterns, initial movement dynamics, and EMD by measuring StartReact responses through a combination of EMG and motion capture. Our findings reveal a significant reduction in EMD in LAS compared to MAS trials, suggesting that enhanced RS drive facilitates faster movement initiation following muscle activation. Additionally, distinct differences in muscle activation and movement profiles were evident between LAS and MAS trials. These novel findings suggest that RS drive not only accelerates muscle activation – consistent with the StartReact effect—but also plays a crucial role in expediting the mechanical transition to movements. Consequently, the RS system facilitates accelerated and enhanced motor responses that are essential for fast, reactive, and ballistic movements.

The influence of descending motor drive on the onset of muscle activation has been explored in numerous studies (Akalu et al., 2023). In a recent study, we mapped reticulospinal drive across the same set of muscles examined in the present work (Eilfort et al., 2025). Our findings demonstrated that – although RS drive was present in all assessed muscles – its magnitude varied significantly across muscles of the upper and lower extremities as measured by the StartReact paradigm (Eilfort et al., 2025). Specifically, a proximal-distal gradient in RS drive was observed in the upper extremities, primarily driven by low RS input to the finger abductor. In contrast, no such proximal-distal gradient was present in the lower extremities. Additionally, distinct patterns of RS innervation were evident between flexors and extensors. In the upper extremities, RS drive was stronger to flexors than to extensors, whereas the opposite pattern was evident in the lower extremities, where greater RS input was directed to extensors than flexors. In contrast to the reported effects RS drive on premotor reaction time, its influence on other aspects of movement initiation remains poorly understood. A more profound understanding of how RS drive affects muscle recruitment patterns and movement initiation is crucial to fully elucidate the contribution of the RS system to rapid movements, such as those involved in protective responses and various athletic activities.

Earlier observations suggest that motor responses to LAS are accelerated, while the overall characteristics of EMG and movement patterns remain largely preserved compared to voluntary, MAS-triggered movements (Valls-Solé et al., 1999). However, more recent evidence indicates that EMG activity patterns are distinct between movements triggered by loud vs. control tones, particularly within the initial 50 to 100 ms following EMG onset (Walker et al., 2024; Škarabot et al., 2022). These studies reported enhanced EMG activity in muscles such as the biceps brachii and vastus lateralis during LAS trials compared to those elicited by non-startling stimuli. In line with this, our results demonstrate markedly enhanced muscle activity in LAS trials during the initial phase of activation. This is evident by enhanced root mean square (RMS) values and higher peak EMG amplitudes during movements triggered by LAS compared to MAS (Škarabot et al., 2022; Castellote and Kofler, 2018). Additionally, reduced latencies of EMG peak amplitudes upon LAS indicates a more rapid and robust muscle activation in response to LAS than MAS. The enhanced muscle activity observed in LAS trials may reflect elevated motor unit discharge rates, as previously demonstrated using high-density EMG recordings (Škarabot et al., 2022). These early-phase changes in EMG activity strongly suggest modulation by descending motor drive resulting in accelerated premotor reaction times (Walker et al., 2024). Prior studies have shown that the initial burst of motor activity in response to startling acoustic stimuli is predominantly mediated by the RS system (Tapia et al., 2022; Neumann et al., 2025). Overall, these findings suggest that the marked differences in initial EMG activity are primarily mediated by subcortical motor centers, most notably the RS system.

The findings of this study indicate that, in addition to altered EMG activity, angular displacements were also enhanced and occurred earlier in LAS compered to MAS trials within the StartReact paradigm. This was reflected in increased RMS and peak amplitude of the angular displacement data, along with shorter latencies to peak displacement. Previous studies have shown that startling acoustic stimuli can increase both the rate of force development and overall force output (Škarabot et al., 2022; Anzak et al., 2011; Fernandez-Del-Olmo et al., 2014). These effects have been suggested to be primarily driven by enhanced motor unit recruitment and elevanted discharge rates, which are likely facilitated by LAS-induced RS drive. The concurrent increases in RMS and peak amplitudes in both EMG and angular displacement suggest a more rapid and forceful neuromuscular response under LAS conditions, likely resulting from enhanced RS drive. Notably, differences in movement performance between LAS vs. MAS trials seem to be mainly based on the accelerated angular displacement, leading to earlier occurrence of the peak amplitude. In contrast, movement amplitudes across all tasks varied by less than 1 degree between LAS vs. MAS trials, consistent with previous findings reporting similar movement amplitudes between MAS-and LAS-induced movements (Valls-Solé et al., 1999; Carlsen et al., 2004).

The influence of descending motor drive on electromechanical coupling is poorly understood. Our findings demonstrate that enhanced RS drive not only shortens reaction time for muscle activation, but also significantly reduces EMD—the time interval between muscle onset and movement initiation. Across all 14 muscles, EMD was shortened by an average of 6 ms in LAS compared to MAS trials. Although EMD shortening was observed in the majority of tasks, the magnitude of this effect varied, with a few tasks showing minimal or no change. A previous study using the StartReact paradigm reported no EMD differences between startling and non-startling trials for the vastus lateralis and vastus medialis (Škarabot et al., 2022), which aligns with our findings of absent EMD differences in this muscle. The most pronounced shortening in EMD in our data was observed in the shoulder extensor. While the overall shortening of EMD in LAS trials was robust, the lack of statistically significant effects in individual muscles may be attributed to limited statistical power after correction for multiple comparisons. Despite this, the mapping approach employed in this study provides compelling evidence for a global effect of RS drive on EMD.

Collectively, these findings suggest that enhanced RS drive supports rapid, explosive movements via two complementary mechanisms: (1) a substantial reduction in premotor RT, reflected in earlier EMG onset (Eilfort et al., 2025), and (2) a shortening of the EMD, which accelerates the mechanical execution of movement. These dual effects highlight the critical contribution of the RS system to efficient neuromuscular performance during high-speed motor actions.

## 5 Limitations

This study measured EMD without differentiating between its electrochemical and mechanical components. Future research could benefit from incorporating mechanomyographic (MMG) assessments, which are able to disentangle these components and provide deeper insights into the mechanisms underlying EMD shortening in response to enhanced RS drive (Orizio, 1993). Additionally, the use of high-density EMG would allow to investigate the impact of descending motor drive on motor unit behavior including motor unit recruitment and discharge patterns. Such approaches would help elucidate the specific physiological processes through which the RS system facilitates accelerated muscle activation and movement initiation.

## 6 Conclusion

In summary, this study demonstrates distinct changes in EMG activation patterns, movement initiation, and EMD when comparing movements that are primarily driven by the corticospinal system (MAS) to those predominately mediated by the RS system (LAS). Our findings suggest that the RS system facilitates more rapid movement initiation through two complementary mechanisms: (1) accelerating the onset of electrical muscle activation and (2) reducing the EMD. These effects likely reflect RS-mediated modulation of motor unit recruitment strategies, potentially influencing the type and timing of muscle fiber activation during movement. Understanding how RS motor drive influences not only premotor reaction time but also the electromechanical coupling phase is critical for a comprehensive understanding of its role in movement initiation. These insights are relevant to various research fields, including sports science, neurorehabilitation and fall prevention, where the ability to produce rapid, intense motor responses is critical.

## Data Availability

The original contributions presented in the study are included in the article/Supplementary material, further inquiries can be directed to the corresponding author.

## Supplementary material

SUPPLEMENTARY FIGURE 1 EMG activity patterns in response to loud (LAS; blue) and moderate acoustic stimuli (MAS; red) across individual tasks. **(A)** Grand average EMG traces (*n* = 29 participants) for each task and LAS/MAS trials from −50 to 200 ms relative to muscle onset. **(B)** EMG root mean squares (RMS) were significantly higher in LAS trials compared to MAS trials for shoulder extension and flexion, elbow extension and flexion, and finger abduction. **(C)** EMG peak amplitudes were significantly enhanced in LAS vs. MAS trials for shoulder extension and flexion, elbow extension and flexion, wrist extension and flexion, finger abduction, knee flexion, ankle plantarflexion and dorsiflexion, and toe extension. **(D)** Peak EMG responses occurred earlier in LAS compared to MAS trials for shoulder flexion, finger abduction, hip extension and flexion, knee extension, and ankle plantarflexion. **B–D** depict medians +/− interquartile ranges of all participants (*n* = 29) from −50 to 200 ms relative to muscle onset (**p* < 0.05, ***p* < 0.01, ****p* < 0.001).

SUPPLEMENTARY FIGURE 2 Kinematic characteristics in response to loud (LAS; blue) and moderate acoustic stimuli (MAS; red) across individual tasks. **(A)** Grand average angular displacements (*n* = 29 participants) for each task and LAS/MAS trials from 0 to 500 ms relative to movement onset. **(B)** Root mean squares (RMS) of angular displacements were significantly higher in LAS vs. MAS trials for shoulder extension and flexion, wrist extension and flexion, knee extension and flexion, and toe extension. **(C)** Peak angular displacements in LAS and MAS trials revealed no significant differences on the single muscle level. **(D)** Timing of peak angular displacement in LAS and MAS trials did not show significant differences in single muscles. **B–D** depict medians +/− interquartile ranges of all participants (*n* = 29) from 0 to 500 ms relative to movement onset (**p* < 0.05).
