# Supplementary figures and images for: Reticulospinal modulation of muscle activation and electromechanical coupling: evidence from the StartReact paradigm
Source: Front Hum Neurosci. 2025 May 21;19:1610211. doi: 10.3389/fnhum.2025.1610211 (PMC12135682; doi:10.3389/fnhum.2025.1610211)

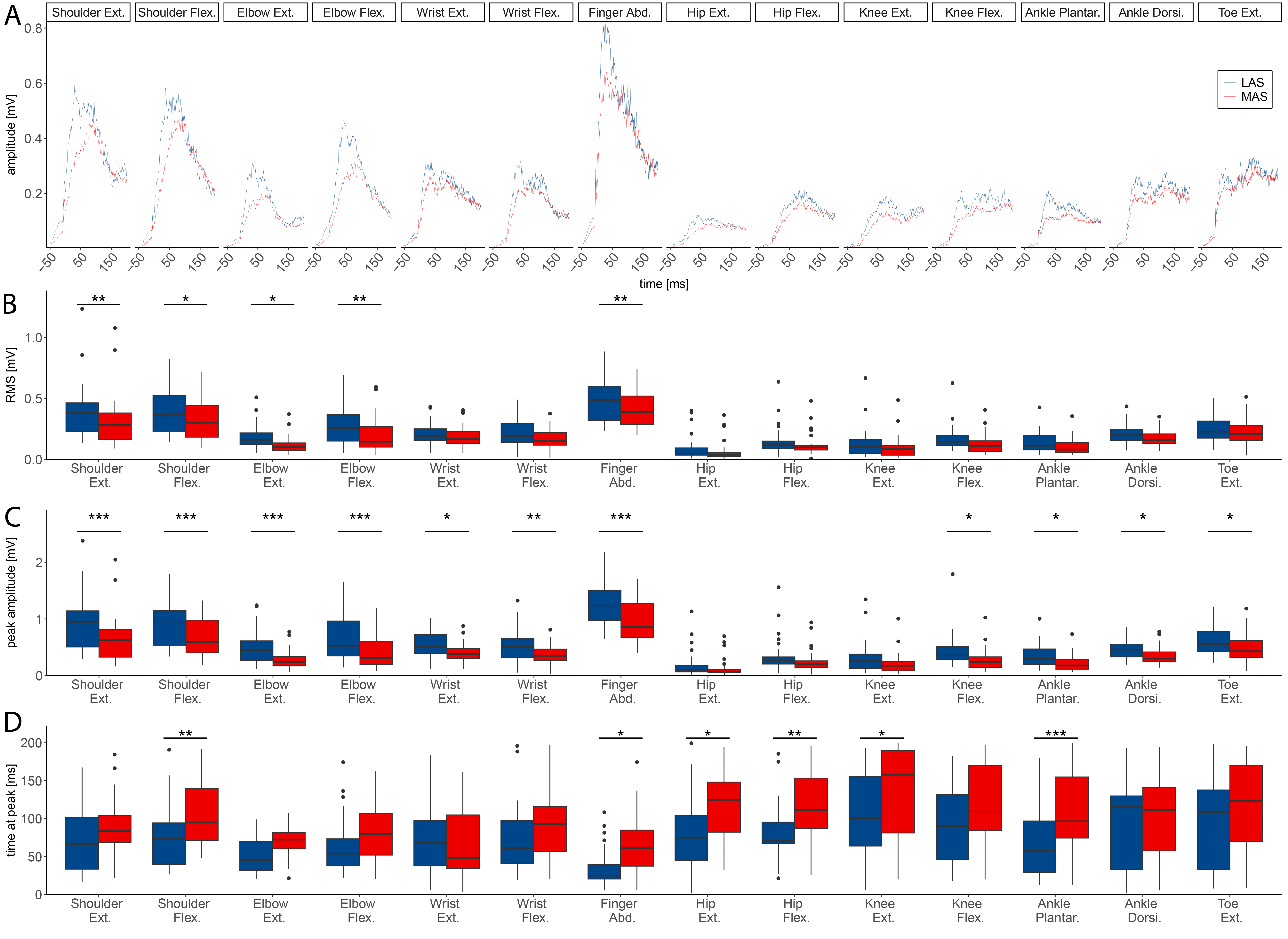

Supplement: SUPPLEMENTARY FIGURE 1 — EMG activity patterns in response to loud (LAS; blue) and moderate acoustic stimuli (MAS; red) across individual tasks. (A) Grand average EMG traces (n = 29 participants) for each task and LAS/MAS trials from −50 to 200 ms relative to muscle onset. (B) EMG root mean squares (RMS) were significantly higher in LAS trials compared to MAS trials for shoulder extension and flexion, elbow extension and flexion, and finger abduction. (C) EMG peak amplitudes were significantly enhanced in LAS vs. MAS trials for shoulder extension and flexion, elbow extension and flexion, wrist extension and flexion, finger abduction, knee flexion, ankle plantarflexion and dorsiflexion, and toe extension. (D) Peak EMG responses occurred earlier in LAS compared to MAS trials for shoulder flexion, finger abduction, hip extension and flexion, knee extension, and ankle plantarflexion. B–D depict medians +/− interquartile ranges of all participants (n = 29) from −50 to 200 ms relative to muscle onset (*p < 0.05, **p < 0.01, ***p < 0.001). [file Image_1.TIF]

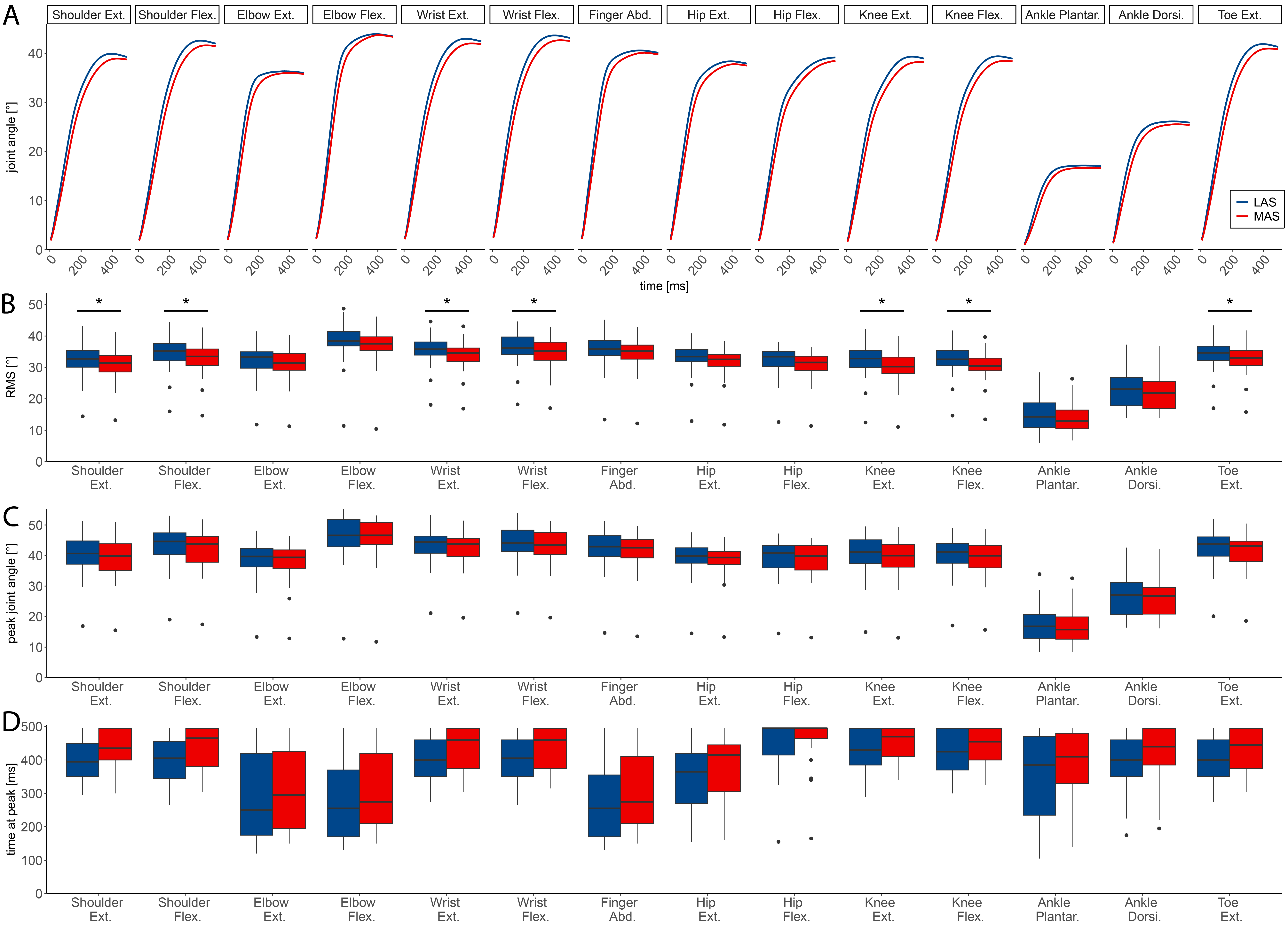

Supplement: SUPPLEMENTARY FIGURE 2 — Kinematic characteristics in response to loud (LAS; blue) and moderate acoustic stimuli (MAS; red) across individual tasks. (A) Grand average angular displacements (n = 29 participants) for each task and LAS/MAS trials from 0 to 500 ms relative to movement onset. (B) Root mean squares (RMS) of angular displacements were significantly higher in LAS vs. MAS trials for shoulder extension and flexion, wrist extension and flexion, knee extension and flexion, and toe extension. (C) Peak angular displacements in LAS and MAS trials revealed no significant differences on the single muscle level. (D) Timing of peak angular displacement in LAS and MAS trials did not show significant differences in single muscles. B–D depict medians +/− interquartile ranges of all participants (n = 29) from 0 to 500 ms relative to movement onset (*p < 0.05). [file Image_2.TIF]
